# Human Cytomegalovirus Genomes Survive Mitosis via the IE19 Chromatin-Tethering Domain

**DOI:** 10.1128/mBio.02410-20

**Published:** 2020-09-29

**Authors:** Shelby M. Lyon, Kristen D. Yetming, Christina Paulus, Michael Nevels, Robert F. Kalejta

**Affiliations:** a Institute for Molecular Virology, University of Wisconsin–Madison, Madison, Wisconsin, USA; b McArdle Laboratory for Cancer Research, University of Wisconsin–Madison, Madison, Wisconsin, USA; c Biomedical Sciences Research Complex, University of St. Andrews, St. Andrews, United Kingdom; St. Jude Children's Research Hospital

**Keywords:** cancer, chromatin, herpes, latency, mitosis, transcription

## Abstract

The genomes of DNA tumor viruses regain nuclear localization after nuclear envelope breakdown during mitosis through the action of a viral protein with a chromatin-tethering domain (CTD). Here, we report that the human cytomegalovirus (HCMV) genome is maintained during mitosis by the CTD of the viral IE19 protein. Deletion of the IE19 CTD or disruption of the IE19 splice acceptor site reduced viral genome maintenance and progeny virion formation during infection of dividing fibroblasts, both of which were rescued by IE19 ectopic expression. The discovery of a viral genome maintenance factor during productive infection provides new insight into the mode of HCMV infection implicated in birth defects, organ transplant failure, and cancer.

## Abstract (precis)

Human cytomegalovirus (HCMV) is the leading infectious cause of birth defects, represents a serious complication for immunocompromised HIV/AIDS and organ transplant patients, and contributes to both immunosenescence and cardiovascular diseases. HCMV is also implicated in cancers such as glioblastoma multiforme (GBM) and infects *ex vivo*-cultured GBM tumor cells. In dividing tumor cells, the genomes of DNA tumor viruses regain nuclear localization after nuclear envelope breakdown during mitosis. This mitotic survival is mediated by a viral protein with a chromatin-tethering domain (CTD). Here, we report that the HCMV genome is maintained in dividing fibroblasts by the CTD of the viral IE19 protein. The discovery of a viral genome maintenance factor during productive infection could help explain viral genome dynamics within HCMV-positive tumors as well as during latency.

## INTRODUCTION

Viruses modify the host cell cycle to induce or synchronize cells in phases that optimally support infection (1). Human cytomegalovirus (HCMV) infection of primary human fibroblasts in the G_0_ or G_1_ phase drives them to the G_1_/S border but prevents them from progressing through the S phase (2–7). Under these conditions, productive infection initiates immediately and is highly efficient. The mechanisms of the rapid initiation of the productive cycle in G_0_/G_1_ cells and their synchronization at the G_1_/S border are reasonably well understood and include the stimulation of viral transcription by the viral pp71 protein, the stimulation of cell cycle progression by pp71 and vCdk UL97, and the G_1_/S arrest instituted by the viral IE2 protein (4, 5, 8–11).

When HCMV infects cells already in the S phase, initiation of the productive cycle is delayed until the infected cell doubles its DNA content, traverses the S and G_2_ phases, completes mitosis, and enters G_0_/G_1_ (12, 13). Productive replication in G_2_ is inefficient and in mitosis causes mitotic catastrophes resulting in abortive, nonproductive, cytocidal infections (14, 15). The mechanism of the delay in the productive replication cycle in S-phase cells involves the interaction of cyclin A2 with the viral pp150 protein to suppress productive-phase viral transcription (16). As infected cells traverse mitosis, cyclin A2 is naturally degraded, and immediate early (IE) transcription initiates in the subsequent G_0_/G_1_ phase of the daughter cells, driving the delayed initiation of productive infection (15). Thus, HCMV devotes multiple mechanisms to synchronization of infected cells in favorable cell cycle phases while preventing cells that are actively replicating virus from entering mitosis, where mitotic catastrophes limit productive replication (14, 15).

While the general mechanisms that HCMV uses to modulate the cell cycle have been solved or are under study, the mechanisms that allow the virus infecting an S-phase cell to initiate productive replication after mitosis have largely been ignored. During mitosis, the nuclear envelope breaks down, and two independent, complete sets of cellular chromosomes are pulled to opposite poles by kinetochore microtubules attached to chromosomal centromeres. New nuclear envelopes then reform around each set of bundled chromosomes. Because viral genomes (including HCMV) lack a centromere, they cannot be reincorporated into daughter nuclei in this fashion. Early work, however, determined that essentially identical percentages of cells express IE proteins at 24 h after HCMV infection regardless of whether the cells were infected in G_0_/G_1_ or S phase (12). This result strongly suggested that in S-phase-infected cells, the viral genome is maintained in the nucleus through mitosis, yet no studies have investigated mechanisms through which this process may occur during these delayed yet productive infections.

For other viruses, mechanisms mediating the nuclear retention of their genomes during mitosis are well established. Retroviruses integrate their genomes into host chromosomes (17). The DNA tumor viruses human papillomavirus (HPV), Epstein-Barr virus (EBV), and Kaposi’s sarcoma-associated herpesvirus (KSHV) maintain their extrachromosomal genomes by using a virally encoded protein to tether them to cellular chromatin (18–20). The HPV E2, EBV EBNA1, and KSHV LANA proteins each contain carboxy-terminal domains that bind sequence specifically to their respective viral genomes (21–25) and amino-terminal domains that bridge and tether the complex to cellular chromatin. The HPV E2 transactivation domain interacts with cellular chromatin-associated proteins (26–28), EBV EBNA1 domains A and B bind to AT-rich cellular DNA and cellular chromatin-associated proteins (29, 30), and the chromatin-binding domain of KSHV LANA binds histones H2A and H2B on cellular or viral genomes (31, 32).

The HCMV *UL123* gene encodes a defined chromatin-tethering domain (CTD) at the 3′ end of its exon 4 (33, 34) that is dispensable for productive replication in asynchronous fibroblast cells (35, 36). In the context of the 72-kDa IE1 protein, the major protein encoded by *UL123*, the CTD associates with the acidic patch of nucleosomes and mediates the colocalization of IE1 with condensed cellular chromosomes similar to KSHV LANA (37, 38). While the *UL123* CTD has long been hypothesized as a viral genome tether (33, 36, 37, 39, 40), the function of this domain has never been tested in mitotic cells.

Like the tethering proteins described above, IE1 dimerizes. The crystal structure of the core domain of rhesus CMV (RhCMV) IE1 (analogous to amino acids 27 to 379 of the 491-amino-acid IE1 protein of HCMV) revealed an antiparallel dimer (41). Dimerization of a truncated fragment of Myc epitope-tagged HCMV IE1 (amino acids 1 to 382) to truncated fragments of FLAG epitope-tagged HCMV IE1 (amino acids 1 to 382 or 1 to 377) was observed by coimmunoprecipitation with Western blotting (41). In the crystal structure, the dimerization interface encompassed the entire length of the core domain and was mainly hydrophilic. A subsequent report (42) used modeling based on the RhCMV IE1 structure to identify five residues within the core domain (K238, Q252, K300, L311, and R325) that provide the strongest effects on predicted binding affinity for the IE1 dimer. Based on alignment (43), the HCMV IE1 equivalent residues are K223, N237, N285, M296, and R310. How dimerization affects IE1 function, or whether other *UL123*-encoded protein isoforms also dimerize, has not been explored.

EBV and KSHV CTDs support viral latency, and there is interest in the role of *UL123* exon 4 during HCMV latency (39). Unfortunately, no system is currently available to assay the maintenance of latent HCMV genomes through mitosis. Therefore, we tested the requirement of the CTD for the only documented instance of the virus surviving mitosis: the infection of fibroblasts residing in the S phase of the cell cycle. We show that the CTD, in the context of an alternatively spliced *UL123* transcript encoding a monomeric protein isoform designated IE19, helps the viral genome to survive mitosis so that the viral genome can initiate the cascade of productive-phase gene expression in daughter cells entering G_1_ phase after completing mitosis.

## RESULTS

### S-phase-infected cells must pass through mitosis to support productive-phase viral gene expression.

**FIG 1:**
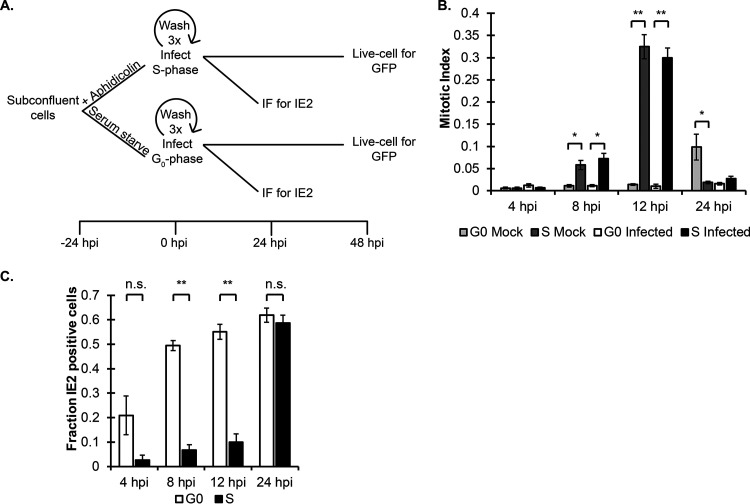
Human cytomegalovirus (HCMV) immediate early (IE) gene expression is silenced after S-phase infection until cells pass through mitosis. (A) Experimental timeline for synchronization via aphidicolin treatment or serum starvation, followed by infection and analysis. (B) Mitotic index was determined after media were supplemented with Hoechst at 4 μg/ml and cells were imaged at the stated time points after release from synchronization. The fraction of cells with condensed chromatin or split nuclei was calculated for at least 1,000 cells and is presented as the mean ± SD for each time point (*n* = 3). (C) Cells synchronized in G_0_ or S were infected at an MOI of 1 and washed to release. Cells were fixed at the indicated time points for analysis by IF with antibodies for IE2 and staining with Hoechst. Data are the means and SD for each time point (*n* = 3). (B and C) *, *P* < 0.05; **, *P* < 0.01; n.s., not significant (*P* > 0.1).

We confirmed previous reports that G_0_/G_1_ fibroblasts infected with HCMV initiate IE gene expression quickly, while S-phase-infected fibroblasts initiate IE gene expression only after a pronounced delay (12). Asynchronous, subconfluent fibroblasts were synchronized either in G_0_ by serum starvation or in S phase by aphidicolin, released from the block, and then infected (Fig. 1A). Released and mock-infected G_0_ cells entered mitosis after a substantial delay (∼24 h), whereas infected cells did not (Fig. 1B), all as expected. Released S-phase cells entered mitosis much faster (∼12 h), whether mock or HCMV infected (Fig. 1B), also as expected, indicating the validity of our synchronization and release protocols. HCMV-infected G_0_ cells were IE2 positive as early as 4 h postinfection (hpi), the earliest time point examined, indicating that they initiated the productive replication cycle, and they achieved essentially maximum percentages of IE2-positive cells by 8 hpi (Fig. 1C). In contrast, HCMV-infected S-phase cultures failed to show substantial percentages of IE2-positive cells as late as 12 hpi, but by 24 hpi they had achieved a percentage of IE2-positive cells indistinguishable from that of the G_0_ infected cultures (Fig. 1C). We conclude, like others before us, that IE gene expression is delayed in S-phase-infected cells until they traverse mitosis and their daughter cells enter the subsequent G_1_ phase (12).

**FIG 2:**
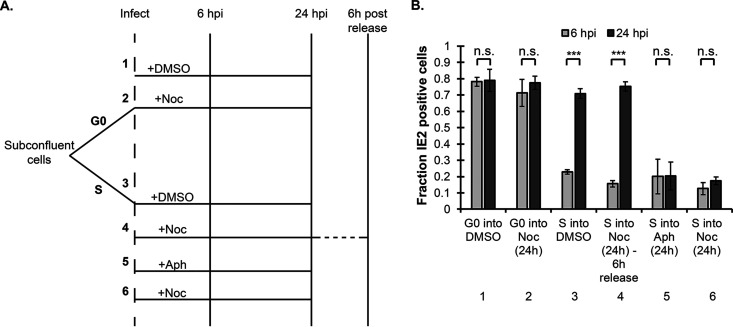
S-phase cells infected with HCMV must pass through mitosis to gain competency for IE gene expression. (A) Experimental timeline for the six infection conditions in panel B. Cells were synchronized with either aphidicolin treatment or serum starvation, followed by infection and supplementation with media containing DMSO, nocodazole, or aphidicolin for 24 h. Cells were fixed at 24 hpi for IE2 and Hoechst analysis by IF. In condition 4, cells were washed at 24 hpi and released for 6 h (30 hpi) before fixing. (B) Fraction of IE2-positive cells was calculated for each condition and is presented as the mean and SD (*n* = 3). ***, *P* < 0.001; n.s., not significant (*P* > 0.1).

We next tested whether a lengthy delay is sufficient to make viral genomes competent for transcription during an S-phase infection, or whether passage through mitosis is required, by infecting S-phase cells synchronized with aphidicolin, releasing them into either dimethyl sulfoxide (DMSO) (to permit cell cycle progression), aphidicolin (to maintain S-phase arrest), or nocodazole (to synchronize cells in mitosis) for 24 h, and monitoring IE2 expression (Fig. 2A). S-phase cells infected and released into DMSO synthesized IE2 (Fig. 2B, lane 3), but those released into aphidicolin (Fig. 2B, lane 5) or nocodazole (Fig. 2B, lane 6) did not. However, when infected S-phase cells were released into nocodazole for 24 h and then subsequently released from the nocodazole block for 6 h (to allow passage through mitosis), IE2 was expressed to a level indistinguishable from that in infected cells directly released from aphidicolin into DMSO (Fig. 2B, compare lanes 4 and 3). Nocodazole itself had no effect on IE2 production in G_0_-infected cells that arrested at the G_1_/S border and never reached mitosis (Fig. 2B, compare lanes 1 and 2), indicating that the drug itself is not inhibitory to viral gene expression unless it arrests cells in mitosis. From these experiments, we conclude that S-phase-infected cells must pass through mitosis to gain competency for viral gene expression in the subsequent G_1_ phase.

### CTD-deficient HCMV displays reduced gene expression, genome levels, and productive replication after S-phase infection compared to the wild type.

**FIG 3:**
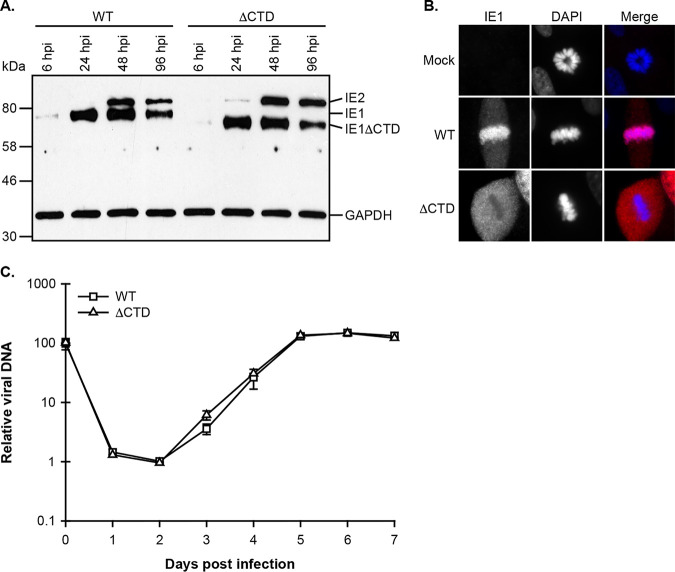
Characterization of CTD-deficient IE1 mutant Towne virus. MRC-5 cells were infected with wild-type (WT) or CTD-deficient (ΔCTD) virus. (A) At the indicated times postinfection, whole-cell extracts were prepared and subjected to Western blot analysis with antibodies against HCMV IE1 and IE2 (MAB810R) and cellular GAPDH. (B) At 48 hpi, cells were fixed and stained with anti-cytomegalovirus IE1 antibody (ab30924) and DAPI (4′,6-diamidino-2-phenylindole). Representative images are shown in grayscale with a color merge. (C) Every 24 h, viral replication was assessed by qPCR-based relative quantification of HCMV DNA from culture supernatants with primers specific for the viral UL54 promoter sequence. Data are means ± SD (*n* = 3).

S-phase-infected cells must pass through mitosis before initiating IE gene expression in the subsequent G_1_ phase at levels equivalent to those in G_0_-infected cells. This suggests that viral genomes are present after mitosis at levels comparable to those prior to mitosis and therefore that viral genomes are retained in the nucleus during mitosis, perhaps by a dedicated mechanism. We tested if such a putative mechanism utilized the *UL123* CTD. We created a CTD-deficient (ΔCTD) recombinant in the Towne strain of HCMV that contains a stop codon at IE1 amino acid position 476, similar to one previously described (35). Our Towne ΔCTD recombinant produces a C-terminally truncated IE1 protein smaller than full-length IE1 (Fig. 3A) that lacks the CTD and fails to associate with mitotic chromosomes (Fig. 3B) but grows with wild-type (WT) kinetics in G_0_ cells (Fig. 3C), all identical to the published virus (35).

**FIG 4:**
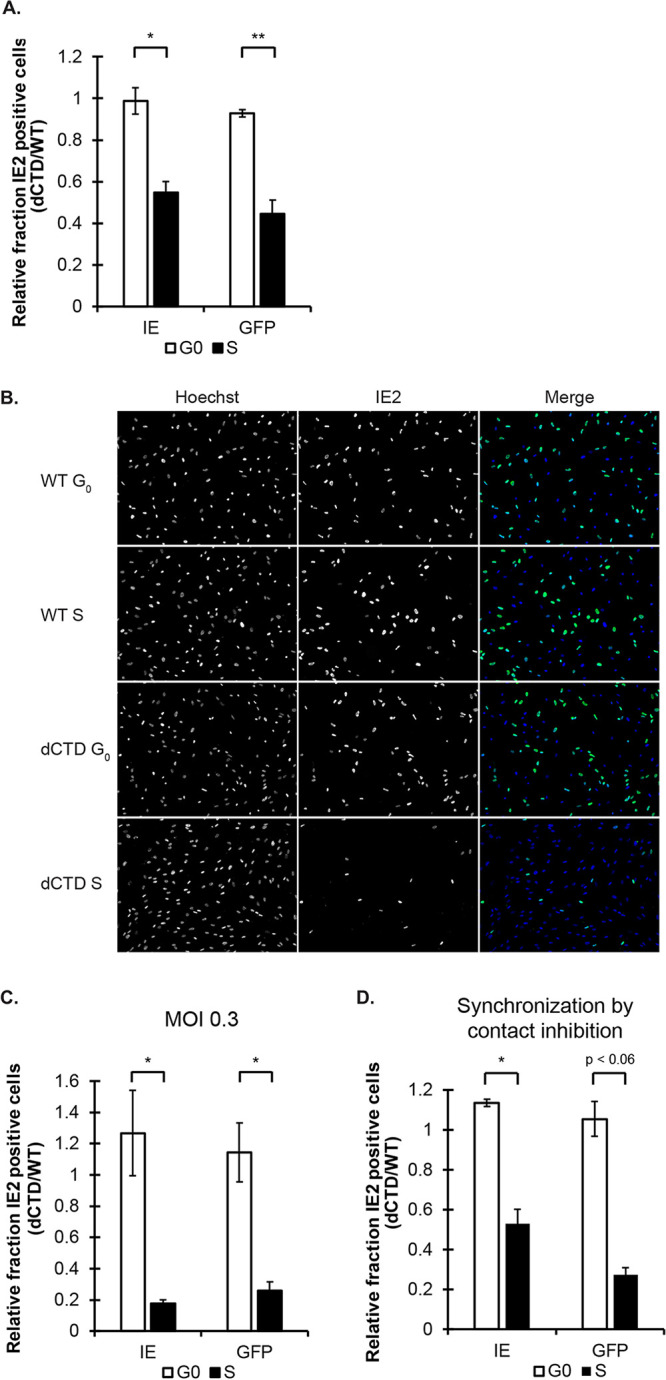
HCMV gene expression is reduced after S-phase infection with a CTD-deficient virus. (A) The fraction of IE2-positive cells was calculated at 24 hpi and the fraction of GFP-positive cells was calculated at 48 hpi following G_0_- or S-phase infection at an MOI of 1. Data are mean IE- and GFP-positive ΔCTD fractions relative to WT fractions ± SD (*n* = 3). (B) Cells synchronized in G_0_ or S were infected at an MOI of 1 and washed to release. Cells were fixed at 24 hpi for analysis by IF with antibodies for IE2 and staining with Hoechst. Representative images are provided in grayscale alongside Hoechst and a color merge. *n* = 3. (C and D) IE2- and GFP-positive fractions were calculated at 24 h and 48 h, respectively, after G_0_- or S-phase infection at an MOI of 0.3 (C) and after S-phase synchronization by contact inhibition and release instead of aphidicolin treatment (D). Data are mean IE- and GFP-positive ΔCTD fractions relative to WT fractions, with SD (*n* = 3). (A, C, and D) *, *P* < 0.05; **, *P* < 0.01; n.s., not significant (*P* > 0.1).

We saw no differences in the fraction of IE2-positive cells between WT and the ΔCTD virus during G_0_ infection (Fig. 4A to D). However, the ΔCTD virus displayed a significantly reduced fraction of IE2-positive cells compared to WT virus after S-phase infection (Fig. 4A to D). The magnitude of the defect was exacerbated at a 3.3-fold-lower multiplicity of infection (Fig. 4C), and the defect was observed using two independent methods to synchronize cells in the S phase: aphidicolin (Fig. 4A to C) or contact inhibition and release (Fig. 4D). Identical results were obtained when production of green fluorescent protein (GFP), encoded on a transgene, by each virus was used to score viral gene expression (Fig. 4A, C, and D). Transcription of the GFP transgene in HCMV is dependent on IE protein synthesis (44), and therefore, GFP protein accumulation was assayed 24 h after IE2 to ensure that all cells had the opportunity to express GFP. We conclude that in S-phase-infected cells, the *UL123* CTD enhances viral gene expression in the subsequent G_1_ phase after completion of mitosis.

**FIG 5:**
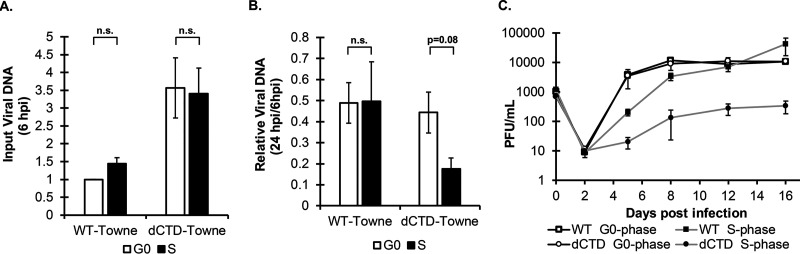
HCMV genome levels and productive infection are reduced after S-phase infection with a CTD-deficient virus. (A) Cells synchronized in G_0_ or S phase were infected for 6 h and harvested for analysis of viral DNA (UL123 exon 3) by qPCR. Data were normalized to cellular DNA (GAPDH) and WT G_0_ and are presented as means and SD (*n* = 3). (B) Cells synchronized in G_0_ or S phase were harvested at 24 hpi for analysis of viral DNA (UL123 exon 3) by qPCR. Data were normalized to GAPDH and input genomes (6 hpi) and are presented as means and SD (*n* = 3). (C) Growth curves were constructed following G_0_- or S-phase infection with the indicated viruses at an MOI of 0.1. Cell-free virus was collected at the stated days postinfection for titration by standard plaque assay. Data are means ± SD (*n* = 3). (A and B) n.s., not significant (*P* > 0.1).

The CTD plays no apparent role in viral gene expression in G_0_ but significantly impacts viral gene expression in cells that pass through mitosis (Fig. 4A), where extrachromosomal DNAs can be lost from the nucleus because of nuclear envelope breakdown (45–47). Therefore, we asked if the CTD helps to maintain viral genome levels in S-phase-infected cells that pass through mitosis. WT virus showed no differences in genome levels between G_0_- and S-phase cells at 6 hpi (Fig. 5A), indicating that the virus entered G_0_- and S-phase cells equivalently, or at 24 hpi (Fig. 5B), indicating that viral genomes that passed through mitosis (S-phase infection) and those that did not (G_0_-phase infection) were maintained equivalently. The ΔCTD virus showed no differences in genome levels at 6 hpi between G_0_- and S-phase cells (Fig. 5A), indicating that this mutant virus also entered G_0_- and S-phase cells equivalently. However, the ΔCTD virus showed lower levels of viral genomes at 24 hpi after S-phase infection than G_0_-phase infection (Fig. 5B). The ΔCTD virus delivered more genomes to cells than did the WT virus (Fig. 5A). There is precedent for a viral mutant to display no growth defect but a higher genome/PFU ratio than WT virus (48). Despite the delivery of ∼3.5-fold more genomes than WT virus to S-phase cells, the ΔCTD virus showed ∼2.5-fold fewer genomes than WT virus after the cells passed through mitosis. Our data indicate that ΔCTD genomes that passed through mitosis were lost more frequently than WT genomes.

The defects in productive-phase transcription and genome level maintenance observed when ΔCTD virus infects S-phase cells extended to defects in progeny virion formation. In G_0_ cells, WT and ΔCTD virus produced infectious progeny in synchrony and to identical levels (Fig. 5C). In S-phase cells, WT virus showed a delay in progeny virion formation (Fig. 5C) similar to the delay observed in viral gene expression (Fig. 1C), but the overall output of infectious progeny eventually matched the level achieved in G_0_ cells. The ΔCTD virus, however, displayed a more pronounced delay in progeny virus formation in S-phase-infected cells, and the overall output of infectious progeny was reduced ∼100-fold from the level achieved in G_0_ cells (Fig. 5C). We conclude that the ΔCTD virus shows pronounced defects in viral gene expression, genome levels, and productive replication after infection of S-phase cells because its genome is lost as the infected cells pass through mitosis.

### An alternatively spliced *UL123* transcript encoding the CTD-containing IE19 isoform is upregulated after S-phase infection.

**FIG 6:**
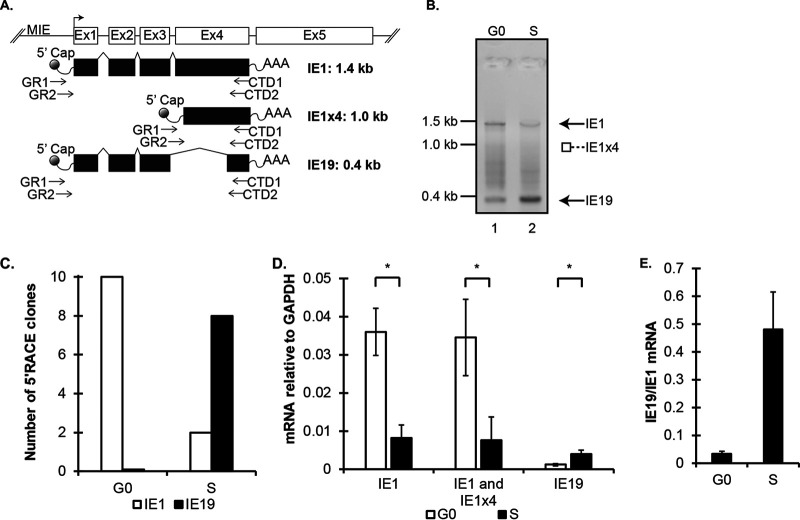
An alternatively spliced *UL123* transcript encoding the CTD-containing IE19 protein is upregulated after S-phase infection. (A) Schematic of the MIE locus showing the splicing of the CTD-containing transcripts IE1, IE1x4, and IE19. Primers used for RLM-RACE are indicated by arrows, and the predicted product size for each transcript is presented on the right. (B) RLM-RACE was conducted on RNA isolated from G_0_- or S-phase cells infected at an MOI of 3 at 6 hpi. The image shows nested PCR products run on a 1% agarose gel and stained with ethidium bromide. Lane 1 is the CTD products from the G_0_-phase sample, and lane 2 is the CTD products from the S-phase sample. (C) Original PCRs for G_0_- and S-phase RLM-RACE samples were cloned. Ten colonies were picked from each and sequenced from the forward and reverse directions to capture the full-length read. All sequences were identical to either IE1 or IE19, and the number of each is presented. (D) Cells synchronized in either G_0_ or S phase and infected at an MOI of 1 were harvested at 6 hpi. RNA was analyzed by qRT-PCR for the indicated transcripts. Data are mean RNA levels relative to cellular GAPDH and SD (*n* = 3). *, *P* < 0.05. (E) The ratio of IE19 transcripts to IE1 transcripts was calculated from the qRT-PCR analysis of RNA from G_0_- or S-phase infections at 6 hpi.

*UL123* must be transcribed during S-phase infections, because the *UL123* CTD is required for genome survival during mitosis. The *UL123* gene produces transcripts encoding IE1, IE1x4, and IE19 (39, 49–51). We utilized RNA ligase-mediated rapid amplification of cDNA ends (RLM-RACE) with primers anchored in the CTD to identify *UL123* transcripts produced following S-phase infection (Fig. 6A). CTD-encoding 1.4-kb and 0.4-kb transcripts consistent with those encoding IE1 and IE19, respectively, were detected (Fig. 6B). We sequenced 10 independent clones derived from the RLM-RACE reaction of S-phase-infected cells and found that two encoded the canonical IE1 transcript while 8 encoded the IE19 transcript (Fig. 6C). No other *UL123* transcripts were identified. For comparison, we sequenced 10 independent clones derived from RLM-RACE reactions from G_0_-infected cells, and all 10 represented the canonical IE1 transcript (Fig. 6C).

To better quantify expression of individual transcript species during infection, we employed quantitative reverse transcription-PCR (qRT-PCR) primer sets specific for IE1 or IE19 that spanned their unique exon 3–exon 4 splice junctions. We also used a primer set in the 5′ end of exon 4 that would detect (but not differentiate between) transcripts encoding IE1 and IE1x4 but would not detect those encoding IE19. Consistent with S-phase restriction of the full cascade of productive-phase gene expression (12), IE1 transcript levels were reduced in S-phase cells compared to G_0_ cells (Fig. 6D). Equivalent results with the IE1 and IE1/IE1x4 primers suggested that a transcript carrying exclusively *UL123* exon 4 was not expressed. IE19 transcripts showed the opposite trend, being higher in S-phase cells than G_0_ cells (Fig. 6D). Importantly, the ratio of IE19 to IE1 in S-phase cells was 10-fold higher than in G_0_ cells (Fig. 6E). Our results indicate that transcripts encoding IE19 are significantly more abundant in S-phase cells than G_0_ cells and implicate IE19 as the CTD-containing protein with the potential to mediate nuclear genome maintenance during S-phase infections.

### IE19-deficient HCMV displays reduced gene expression, genome levels, and productive replication after S-phase infection compared to the wild type.

**FIG 7:**
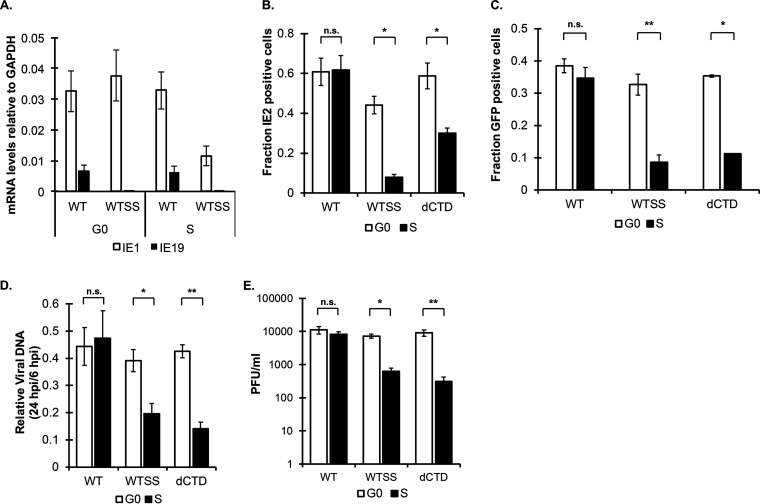
IE19-deficient HCMV displays reduced gene expression, genome levels, and productive replication after S-phase infection. (A) RNA harvested from either G_0_- or S-phase infections with WT or WTSS Towne at 24 hpi was analyzed by qRT-PCR for IE19. Data are mean RNA levels relative to cellular GAPDH and SD (*n* = 3). (B) The fraction of HCMV-positive cells after infection in G_0_ or S phase with the indicated viruses at an MOI of 1 was measured by IF for IE2 and Hoechst at 24 hpi. Data are means and SD (*n* = 3). (C) The fraction of HCMV-positive cells after infection in G_0_ or S phase with the indicated viruses at an MOI of 1 was measured by live-cell imaging for GFP and Hoechst at 48 hpi. Data are means and SD (*n* = 3). (D) DNA harvested at 24 hpi from G_0_- or S-phase infections with the indicated viruses at an MOI of 1 was analyzed by qPCR for viral DNA (UL123 exon 3). Data are presented as mean DNA levels relative to cellular GAPDH, with SD (*n* = 3). (E) Cell-free virus was collected at 8 dpi (days postinfection) after G_0_- or S-phase infection with the indicated viruses at an MOI of 0.1. Endpoint titers were determined by standard plaque assay. Data are means and SD (*n* = 3). (B to D) *, *P* < 0.05; **, *P* < 0.01; n.s., not significant (*P* > 0.1).

We tested if IE19 contributed to the nuclear retention of viral genomes during mitosis using an IE19-deficient recombinant in the Towne strain of HCMV (WTSS) containing a silent mutation in the 3′ splice site of IE19 (50). In G_0_- or S-phase cells, the WTSS virus failed to generate IE19-encoding mRNAs (Fig. 7A). Both the WTSS and the ΔCTD viruses displayed significantly reduced fractions of IE2-positive cells compared to WT virus after S-phase infection (Fig. 7B). Identical results were obtained when production of GFP encoded on a transgene by each virus was used to score viral gene expression (Fig. 7C). WT virus showed no differences in genome levels at 6 or 24 hpi between G_0_- and S-phase cells, and the ΔCTD and WTSS viruses showed no differences in genome levels at 6 hpi between G_0_- and S-phase cells. However, both mutant viruses showed lower levels of viral genomes at 24 hpi after S-phase infection than G_0_-phase infection (Fig. 7D), indicating that ΔCTD and WTSS genomes that passed through mitosis were lost more frequently than WT genomes.

The defects in productive-phase transcription and genome level maintenance observed when ΔCTD or WTSS virus infected S-phase cells extended to defects in progeny virion production. In G_0_ cells, WT, ΔCTD, and WTSS viruses produced infectious progeny to identical levels (Fig. 7E). In S-phase cells, the overall output of infectious progeny for WT virus matched the level achieved in G_0_ cells but was reduced 10-fold for the ΔCTD and WTSS viruses (Fig. 7E). We conclude that the WTSS virus, similarly to the ΔCTD virus, shows pronounced defects in viral gene expression, genome levels, and productive replication after infection of S-phase cells because its genome is lost as the infected cells pass through mitosis in the absence of the CTD of the IE19 protein. Therefore, IE19, with its CTD, facilitates the retention of viral genomes in dividing cells.

### IE19, but not IE1 or IE1x4, complements the deficiencies in gene expression, genome levels, and productive replication displayed by CTD- or IE19-deficient viruses after S-phase infection.

**FIG 8:**
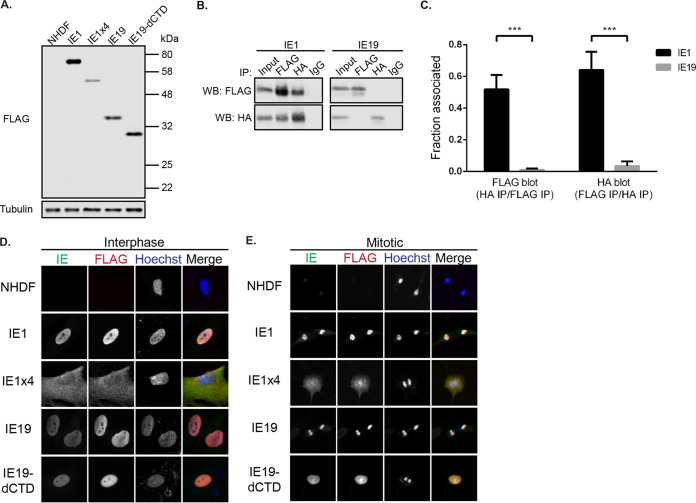
Characterization of fibroblasts expressing IE1, IE1x4, IE19, or IE19ΔCTD. (A) Lysates from equal numbers of cells expressing the indicated protein were harvested and analyzed with the indicated antibodies by Western blotting. Approximate sizes are shown. *n* = 3. (B) Transfected 293T cells expressing 3×FLAG- or HA-tagged IE19 and IE1 proteins were cross-linked with 1% formaldehyde, and lysates were subjected to immunoprecipitation with the indicated antibody, separation by SDS-PAGE, and analysis by Western blotting with antibodies against the FLAG and HA epitopes. *n* = 3. (C) The ratios of signal intensities of HA IP/FLAG IP for the FLAG blots and FLAG IP/HA IP for the HA blots were calculated for IE1 and IE19. Error bars indicate standard deviations. *n* = 3. ***, *P* < 0.001. (D) Cells expressing the indicated proteins were stained for IE1, FLAG, and Hoechst by IF. Representative images are presented in grayscale with a color merge. *n* = 3. (E) Cells expressing the indicated proteins were synchronized with aphidicolin for 24 h and washed to release. At 12 h postrelease, cells were fixed and stained for IE1, FLAG, and Hoechst by IF. Representative images are presented in grayscale with a color merge. *n* = 3.

Finally, we used a complementing cell line approach to determine if IE1, IE1x4, or IE19 expressed in *trans* could complement the S-phase infection defects observed with the ΔCTD and WTSS viruses. Primary normal human dermal fibroblasts (NHDFs) were transduced with lentiviruses encoding N-terminally tagged cDNAs for either IE1, IE1x4, IE19, or IE19ΔCTD (IE19 missing the CTD) and a puromycin resistance gene. Puromycin-resistant populations were selected that expressed the appropriately sized protein encoded by the transduced *UL123* cDNA (Fig. 8A). IE1 migrated as the expected 72-kDa species. IE1x4 migrated at ∼55 kDa, which is comparable to the ∼60-kDa species previously reported (39). IE19 migrated as the expected ∼38-kDa species even though its predicted molecular weight is 19 kDa (51), and IE19ΔCTD migrated at a molecular weight consistent with the artificial truncation. The 38-kDa apparent molecular weight of IE19 is exactly twice its predicted molecular weight, and the DNA tumor virus CTD-containing mitotic maintenance proteins HPV E2, EBV EBNA1, and KSHV LANA all function as dimers (52–56). However, in experiments where we detected dimerization of full-length IE1, we were unable to detect IE19 dimerization (Fig. 8B and C). Differentially epitope (FLAG or HA)-tagged IE1 coimmunoprecipitated the oppositely tagged protein (HA or FLAG, respectively) in transfection assays (Fig. 8B) in an efficient manner (Fig. 8C), but no association was detected between differentially tagged forms of IE19 (Fig. 8B and C). Thus, it is likely that the acidic nature of the amino acid sequence of IE19 causes aberrant migration on SDS-PAGE, as has been detected for other proteins (57–59).

The IE1, IE19, and IE19ΔCTD proteins displayed a diffuse nuclear localization in interphase cells (Fig. 8D), consistent with the presence of the known nuclear localization signal (NLS) encoded within the exon 2 sequences of each cDNA (34). The IE1x4 protein, whose subcellular localization was not analyzed when its existence was reported (39), showed diffuse cytoplasmic staining in interphase cells (Fig. 8D), consistent with the lack of the NLS sequences encoded in exon 2. In mitotic cells, IE1 and IE19 colocalized with cellular chromatin (Fig. 8E), consistent with their identical encoded CTDs. Neither IE19ΔCTD nor IE1x4 colocalized with cellular chromatin in mitotic cells (Fig. 8E), consistent with IE19ΔCTD lacking the CTD and IEx4 localizing to the cytoplasm.

**FIG 9:**
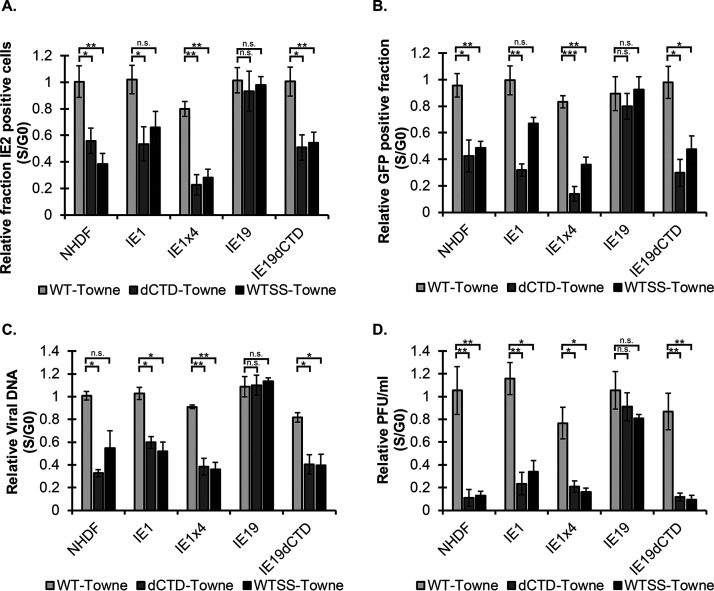
IE19, but not IE1 or IE1x4, complements the deficiencies in gene expression, genome levels, and productive replication displayed by CTD- or IE19-deficient viruses after S-phase infection. (A) Cells expressing the indicated proteins were synchronized in G_0_ or S phase and infected at an MOI of 1 with the indicated viruses. The fraction of HCMV-positive cells was measured by IF for IE2 and Hoechst at 24 hpi. Data are mean fractions of positive cells from S-phase infection relative to G_0_ infection, with SD (*n* = 3). (B) Cells expressing the indicated proteins were synchronized in G_0_ or S phase and infected at an MOI of 1 with the indicated viruses. The fraction of HCMV-positive cells was measured by live-cell imaging for GFP and Hoechst at 48 hpi. (C) DNA harvested at 24 hpi from G_0_- or S-phase infection at an MOI of 1 was analyzed by qPCR for viral DNA (UL123 exon 3) and cellular DNA (GAPDH). Data are mean DNA levels normalized to GAPDH from S-phase infection relative to G_0_ infection, with SD (*n* = 3). (D) Cell-free virus was collected at 8 dpi after G_0_- or S-phase infection with the indicated viruses at an MOI of 0.1 Endpoint titers were determined by standard plaque assay. Data are mean S phase infection titers relative to G_0_ infection, with SD (*n* = 3). (B to D) *, *P* < 0.05; **, *P* < 0.01; ***, *P* < 0.001; n.s., not significant (*P* > 0.1).

Both the ΔCTD and WTSS viruses displayed a significantly reduced fraction of IE2-positive cells compared to WT virus after S-phase infection of the parental NHDF cells, as well as after S-phase infection of NHDFs expressing IE1, IE1x4, or IE19ΔCTD (Fig. 9A), indicating that none of these proteins complemented the defects inherent in the absence of the CTD (ΔCTD virus) or the absence of the IE19 protein (WTSS virus). Similarly, neither IE1, IE1x4, nor IE19ΔCTD was able to complement S-phase-infection defects in GFP expression (Fig. 9B), viral DNA maintenance (Fig. 9C), or infectious-virion production (Fig. 9D) for the ΔCTD or WTSS viruses. However, all known S-phase-infection defects for ΔCTD and WTSS viruses were complemented in IE19-expressing cells. Both the ΔCTD and WTSS viruses displayed IE2 expression (Fig. 9A), GFP expression (Fig. 9B), viral genome maintenance (Fig. 9C), and infectious-progeny production (Fig. 9D) indistinguishable from those of WT virus after infection of IE19-expressing fibroblasts, indicating that IE19 expressed in *trans* complements the defects inherent in the absence of the CTD (ΔCTD virus) or the absence of the IE19 protein (WTSS virus). In total, we conclude that HCMV genomes are maintained through mitosis in productively infected fibroblasts by the CTD of IE19.

## DISCUSSION

Even though they contain identical CTDs, IE1 does not mediate viral genome mitotic maintenance during productive infection of S-phase cells, while IE19 does (Fig. 9). Aside from the missing amino acids, another difference between the two proteins is that IE1 is detected as a dimer and IE19 is not (Fig. 8B and C). IE1 dimerizes through its core domain (41, 42). While the beginning of the core domain (amino acids 25 to 85) is encoded by exon 2 and thus is present in IE19, the majority of the core domain (amino acids 86 to 379) is encoded in the region of exon 4 that is spliced out to make the IE19-encoding mRNA and therefore is not found in the IE19 protein. Of the five residues predicted to drive IE1 dimerization, none is present in IE19. Thus, IE19 would not be predicted to, nor did we observe it to, dimerize (Fig. 8B).

Tethering of viral genomes or chromatin to cellular chromatin requires the tethering protein to simultaneously bind both nucleic acid complexes either directly or indirectly. IE19 lacking the CTD does not associate with cellular chromatin (Fig. 8D), implicating the CTD as the functional domain that might bind to cellular chromatin during the mitotic maintenance of viral genomes. The fact that IE19 is not a dimer and therefore functions with only a single CTD implies that an additional region of the protein other than the CTD is responsible for binding viral DNA directly or indirectly. A postulated viral DNA-associating region could be the acidic domain, as has been speculated for IE1x4 (39), or it could be a different region of the IE19 protein. Perhaps IE1 dimerization, or the folded nature of the core domain, sterically blocks the postulated but as-yet-unidentified functional surface required for viral DNA association. Thus, while IE1 retains the CTD and binds chromatin, perhaps it does not bind viral DNA and as such cannot mediate viral genome maintenance through mitosis.

In addition to its inability to shepherd viral genomes through mitosis, IE1 is a highly immunogenic protein. During the extended nonproductive phase of an S-phase infection where viral immune evasion genes do not appear to be expressed, avoiding immune detection may be paramount to viral survival *in vivo*. Interestingly, eight of the 10 most prominently recognized CTL epitopes in IE1 (60) are absent in IE19. Along with alterations in the presence or sequence of CTL epitopes, maintaining low viral protein levels is an effective immune evasion strategy common to many viruses. Thus, and perhaps not surprisingly, like others before us (49, 50), we have been unable to detect the IE19 protein in HCMV-infected cells, despite numerous attempts with multiple different protein enrichment or stabilization protocols. In the absence of a detectable protein, it remains formally possible that the IE19-coding mRNA functions in a noncoding capacity. However, because all other known examples of viral genome maintenance through mitosis are mediated by viral proteins, we hypothesize that the IE19 protein mediates HCMV genome maintenance through mitosis, despite our inability to detect it with the currently available reagents. We suspect that antibodies with higher affinity or greater specificity would permit detection of the IE19 protein during HCMV infection and that IE19 levels are simply kept as low as possible to avoid immune detection and clearance of the infected cell. Thus, the use of the IE19 protein for viral genome survival through mitosis during S-phase infection, as opposed to larger CTD-containing proteins encoded by *UL123* like IE1 or IEx4, may have emerged not only for functional competency but also to escape immune surveillance.

Viruses other than HCMV for which CTD-mediated extrachromosomal viral genome maintenance during mitosis has been demonstrated are oncogenic. HCMV has been implicated as a cofactor for glioblastoma multiforme (GBM) tumors (61–63). During *ex vivo* infections of primary GBM samples, HCMV productively replicates in some cells, but in others it increases their capacity for self-renewal (64), indicating perhaps that in these dividing cells the virus is being maintained in the absence of progeny virion formation. A mechanism for genome maintenance during mitosis would appear to be required to maintain this type of nonproductive infection, similar to how HPV, EBV, and KSHV genomes are maintained in tumor cells by chromatin tethering in the absence of progeny virion formation. In addition to cancers, HCMV is also implicated in proliferative atherosclerotic diseases such as transplant-associated vasculopathy (65–67). Our data indicate that IE19 activity facilitates the infection of dividing cells by providing a genome retention function during mitosis. Should an inhibitor of IE19 function emerge, it could impede HCMV infection in dividing cells and as such might be useful in combination therapies for the treatment of diseases such as cancers and cardiovascular diseases that may be potentiated by HCMV infection of proliferating cells.

The mitotic maintenance of DNA tumor virus genomes occurs in cells that undergo multiple mitoses, and therefore, the extrachromosomal viral genomes replicate to prevent the dilution of the viral genome that would eventually limit the number of viral genome-positive cells (18, 19). The mitotic maintenance of HCMV genomes occurs in S-phase cells that undergo only one mitosis, after which productive replication initiates, arrests the cell cycle, and eventually kills the cell. Thus, in the absence of a need for infected-cell expansion, we have yet to examine whether the HCMV genome replicates after S-phase infection of fibroblasts.

The mitotic maintenance of DNA tumor virus genomes occurs during nonproductive (persistent or latent) infections. Our demonstration here that HCMV genomes in productively infected cells survive mitosis through a mechanism similar to those utilized by the DNA tumor viruses leads one to consider if such an event also transpires during latency. Currently, it is unclear whether cells latently infected with HCMV divide *in vivo* or *in vitro*, and there are no assays that quantitate latent genome replication or survival through mitosis. However, our demonstration here of a dedicated, IE19-dependent mechanism for viral genome survival through mitosis in differentiated cells makes it possible that an identical or similar mechanism maintains latent genomes in a dividing population of the incompletely differentiated myeloid cells where HCMV maintains latency.

CD34^+^ hematopoietic progenitor cells infected with an HCMV recombinant lacking exon 4 (and therefore lacking IE19 and the CTD) were found to harbor less viral DNA 35 days after infection than WT virus-infected cells (39). However, that study did not quantitate the viral DNA, did not test for actual viral genome replication or maintenance in infected cells that were dividing, and did not control for the potential productive amplification of the WT virus used (the exon 4 mutant is unable to replicate in noncomplementing cell lines and thus could not productively amplify in these experiments).

The major immediate early promoter (MIEP) that directs *UL123* transcription is repressed during HCMV latency (68). Occasionally, transcripts annotated to *UL123* are detected during latency that may be explained by nascent reactivation events, transcriptional noise, or overly high-MOI *in vitro* infections. However, they may also represent CTD-containing transcripts (possibly IE19) making proteins that mediate viral genome replication and/or mitotic maintenance during latency. More work is needed to determine whether IE19 or some other CTD-containing protein mediates viral genome maintenance during HCMV latency.

Finally, the repression of IE1 expression after S-phase infection and its activation in the subsequent G_1_ phase mimic, to some degree, the silencing of IE1 during the establishment and maintenance of latency and the animation of IE1 expression during reactivation to productive replication (69). It remains to be determined whether similar or different mechanisms control *UL123* transcriptional suppression, activation, and splicing during S phase and latent infections.

## MATERIALS AND METHODS

### Cells and infections.

Normal human dermal fibroblasts (NHDFs; Clonetics), human embryonic lung fibroblasts (MRC5; ATCC), and 293T cells were maintained in Dulbecco's modified Eagle medium (DMEM; Sigma) supplemented with 10% (vol/vol) fetal bovine serum (FBS; Sigma) and 1× penicillin-streptomycin with l-glutamine (PSG) (G1146; Sigma). Synchronization by serum starvation was conducted by plating cells at 1 × 10^4^ cells/cm^2^ for 16 h. Cells were washed three times with Dulbecco's phosphate-buffered saline (DPBS; Invitrogen) and incubated for 24 h in low-serum medium (0.1% FBS–DMEM+PSG) before infection. Synchronization by contact inhibition was conducted by feeding confluent cells complete medium daily for 120 h, followed by replating at 2 × 10^4^ cells/cm^2^ for 16 h before infection. Synchronization by aphidicolin treatment was conducted by plating cells at 1 × 10^4^ cells/cm^2^ for 16 h and changing the medium to complete DMEM containing 2 μg/ml aphidicolin for 24 h before infection. Infections with HCMV were conducted in minimal volume for 60 min at 37°C with rocking every 10 min, followed by the addition of fresh medium to normal volume conditions. A multiplicity of infection (MOI) of 1 was used in all experiments unless otherwise specified to avoid the spurious retention of extrachromosomal DNAs (unaided by a viral retention function) that occurs in direct relation to their numbers within individual nuclei. Furthermore, an MOI of 1 generates ∼50% IE-positive cells during G_0_ infections, easily allowing either increases or decreases of IE-positive cells to be quantitated. WT, ΔCTD, and WTSS viruses are in the Towne backbone and express GFP from a simian virus 40 (SV40) promoter. In the ΔCTD virus, a single engineered point mutation converts codon 476 from a glycine codon (GGA) to a stop codon (TGA). The identity of the viruses was confirmed by sequencing of the *UL123* region. The WTSS virus was provided by Jeff Meier and Mark Stinski (University of Iowa) and was described previously (50). Two engineered point mutations in the WTSS virus convert the splice acceptor site within exon 4 that is required to generate IE19 from 5′-CAG AGT-3′ to 5′-CgG AaT-3′. These mutations inhibit splicing but do not affect the amino acid sequence of IE1. The identity of the viruses was confirmed by demonstrating that the WT produces the spliced mRNA encoding IE19 and that WTSS does not (Fig. 7A).

### Inhibitors and antibodies.

Where indicated in the figure legends and on the figures, aphidicolin (2 μg/ml; Sigma) or nocodazole (50 ng/ml; Sigma) was diluted in fresh medium and added to cells. The following antibodies were from commercial sources: anti-glyceraldehyde-3-phosphate dehydrogenase (GAPDH) (ab9485; Abcam), anti-tubulin (DM 1A; Sigma), anti-FLAG (PA1-984B, Thermo), anti-FLAG (M2; Sigma), anti-HA (HA.11, BioLegend), anti-cytomegalovirus for IE1 (ab30924; Abcam), and anti-cytomegalovirus for IE1 and IE2 (MAB810R; Millipore). Monoclonal antibodies against IE1 (1B12) and IE2 (3H9) were described previously (70). Infrared (IR) dye 680- and 800-conjugated secondary antibodies (Li-Cor) were used for Western blotting. Alexa Fluor 488-conjugated secondary antibody (catalog no. A-11017; Invitrogen) and Alexa Fluor 594-conjugated secondary antibody (catalog no. A-11020; Invitrogen) were used for immunofluorescence.

### Transductions.

Lentiviral transduction of fibroblasts was performed as previously described (71). Fibroblasts were transduced with pSin lentiviral vectors expressing IE1, IE19, IE1x4, or IE19ΔCTD, all with 3×FLAG tags on their N termini. IE19ΔCTD was created by truncating IE19 at amino acid 156 and inserting a stop codon. Transduced cells were maintained in complete medium containing 1 μg/ml puromycin (Sigma).

### Coimmunoprecipitations.

293T cells were cotransfected with pSG5 expression vectors encoding 3×FLAG- or HA-tagged IE19 and IE1 alleles using Lipofectamine 2000 (Life Technologies), according to the manufacturer’s instructions. At 2 days posttransfection, the cells were harvested and cross-linked with 1% formaldehyde for 10 min at room temperature. To quench, glycine was added to a final concentration of 125 mM and incubated for 5 min at room temperature. The cross-linked cell lysates were pelleted, washed with cold PBS, and resuspended in 1× cell lysis buffer (Cell Signaling Technologies) supplemented with 1 mM phenylmethylsulfonyl fluoride (PMSF; Cell Signaling Technologies). Samples were sonicated three times on ice for 5 s each and centrifuged at 14,000 × *g* for 10 min at 4°C, and the supernatant (cell lysate) was transferred to a new tube. Cell lysates were incubated with rotation overnight with 2 μg of anti-FLAG (M2), anti-HA.11, or mouse IgG isotype control antibody (I5381; Sigma). Protein A/G magnetic beads (Thermo Scientific) were prewashed with 1× cell lysis buffer plus 1 mM PMSF and then incubated with the immunocomplex for 40 min at room temperature with rotation. The beads were pelleted using a magnetic rack and washed five times with 1× cell lysis buffer plus 1 mM PMSF. For SDS-PAGE, 2× SDS protein sample buffer (125 mM Tris-HCl [pH 6.8], 4% SDS, 20% glycerol, 0.004% bromophenol blue) supplemented with 5% β-mercaptoethanol was added, and the samples were boiled for 10 min prior to separation by SDS-PAGE and analysis by Western blotting. Signal intensities were acquired using Image Studio version 5.2 software (Li-Cor). The ratios of signal intensities of HA IP/FLAG IP for the FLAG blots and FLAG IP/HA IP for the HA blots are presented as means with standard deviations of three biological replicates. An unpaired, two-tailed Student's *t* test was used to determine the statistical difference between IE1 and IE19 (*P* < 0.001).

### Indirect immunofluorescence assays.

Cells were cultured, synchronized, and/or infected for indirect immunofluorescence on a μ-Slide VI 0.4 (80606; Ibidi). At various time points, cells were washed twice with cold PBS and fixed with 1% paraformaldehyde (PFA) either for 30 min at room temperature or overnight at 4°C. Immunofluorescence (IF) was assessed as previously described (10) except to follow manufacturer’s recommendations (AN03 and MV18; Ibidi) for volumes and handling. Images were collected with a Nikon Ti-Eclipse inverted wide-field microscope, taken with a CoolSnap HQ camera, and recorded with Nikon NIS Elements software (v 4.00.03). At least 500 nuclei were counted per condition. Images were processed and background subtracted using NIH FIJI/ImageJ software.

### DNA and mRNA analysis.

For Fig. 3C, viral DNA was isolated from culture supernatants with the DNeasy blood and tissue kit (69506; Qiagen) and quantitated as previously described (72). For all other experiments, total DNA was isolated using a genomic DNA minikit (IB47202; IBI). Total DNA (200 ng) was analyzed by quantitative PCR using iTaq Universal SYBR green Supermix (172-5124; Bio-Rad) on an ABI7900HT real-time PCR system (Applied Biosystems) instrument. Viral genomes were amplified with primers specific to exon 3 of IE1/IE2 (73) or the UL54 promoter region (74), and cellular DNA was amplified with glyceraldehyde-3-phosphate dehydrogenase (GAPDH) primers (75). For transcript analysis, total RNA was isolated using a total RNA minikit (IB47232; IBI) according to the manufacturer's instructions. Total RNA (400 ng) was converted to cDNA using the Maxima H Minus first-strand cDNA synthesis kit (K1681; Thermo Fisher) according to the manufacturer's instructions. Quantitative reverse transcription-PCR (qRT-PCR) was performed as previously described (76) in technical triplicate. Melting curve analysis confirmed the presence of a single PCR product for each primer set. Data were analyzed with SDS 2.4 software (Applied Biosystems), and viral gene expression was normalized to cellular GAPDH using the Δ*C_T_* method, where *C_T_* is threshold cycle (77). Primer sequences are provided in Table 1.

**TABLE 1:** Oligonucleotides used in this study

Primer	Sequence	Reference
qPCR primers		
IE19 forward	5′-GCAGAACTCGAGTCCCCT-3′	This paper
IE19 reverse	5′-TTACTGGTCAGCCTTGCT-3′	This paper
Exon 4 forward	5′-TATACCCAGACGGAAGAGAAAT-3′	39
Exon 4 reverse	5′-CCTTCAGTGCACCCCCTAACTT-3′	39
Exon 3-4 (IE1) forward	5’-TTCCCAGAATTGGCCGAAGAA-3′	This paper
Exon 3-4 (IE1) reverse	5’-CGCACCATGTCCACTCGAAC-3′	This paper
5' RACE GSP1 CTD reverse	5′-CTGGTCAGCCTTGCTTCTAGTCA-3′	This paper
5' RACE GSP2 CTD reverse	5′-CTCAGCACCATCCTCCTCTTCCT-3′	This paper
GAPDH forward	5′-GAGCCAAAAGGGTCATC-3’	75
GAPDH reverse	5′-GTGGTCATGAGTCCTTC-3′	75
Exon 3 forward	5′-CGACGTTCCTGCAGACTATG-3′	73
Exon 3 reverse	5′-TCCTCGGTCACTTGTTCAAA-3′	73
UL54 promoter forward	5′-CACCAAAGACACGTCGTT-3′	74
UL54 promoter reverse	5′-GTCCTTTGCGACCAGAAT-3′	74

Cloning primers		
FLAG-IE1/IE19 forward	5′-CTCACTATAGGGCGAATTCATGGACTACAAAGACCATGACGGTGATTATAAAGATCATGACATCGATTACAAGGATGACGATGACAAGGAGTCCTCTGCC-3’	This paper
FLAG-IE1/IE1x4/IE19 reverse	5′-TTTAATAAGATCTGGATCCTTACTGGTCAGCCTTGCT-3′	This paper
FLAG-IE1x4 forward	5′-CTCACTATAGGGCGAATTCATGGACTACAAAGACCATGACGGTGATTATAAAGATCATGACATCGATTACAAGGATGACGATGACAAGGTGCGGCATAGAATCAA-3’	This paper
FLAG-IE19ΔCTD reverse	5′-TTTAATAAGATCTGGATCCTTAAGAGGCGGTGGGTTC-3′	This paper

### RLM-RACE.

G_0_- or S-phase synchronized cells were infected with WT Towne for 6 h at an MOI of 3. RNA was isolated from 1 × 10^6^ cells using a Total RNA minikit (IB47232; IBI) according to the manufacturer's instructions. Isolated RNA was used directly for RLM-RACE using the GeneRacer kit with SuperScript III RT and TOPO TA cloning kit for sequencing (L150201; Invitrogen), following the manufacturer’s instructions.

### Western blotting.

Cells were lysed in 1% SDS containing 2% β-mercaptoethanol and boiled for 20 min prior to separation by SDS-PAGE and transfer to Optitran membranes (GE Healthcare). Membranes were blocked in 5% bovine serum albumin (BSA) in Tris-buffered saline with Tween 20 (TBST) followed by incubations in primary and secondary antibody (10). Membranes were washed in TBST and imaged with the Odyssey Fc imager (Li-Cor). Images were generated with Image Studio version 5.2 software (Li-Cor).

### Data presentation and analysis.

All graphs present means and standard deviations for three biological replicates. An unpaired, two-tailed Student's *t* test was used to determine the statistical difference between G_0_ and S samples where indicated.
